# Neuroprotective effects of bone marrow Sca-1^+^ cells against age-related retinal degeneration in OPTN E50K mice

**DOI:** 10.1038/s41419-021-03851-0

**Published:** 2021-06-15

**Authors:** Xinna Liu, Mingying Hou, Shiqi Zhang, Yutong Zhao, Qi Wang, Menglu Jiang, Mengxian Du, Zhengbo Shao, Huiping Yuan

**Affiliations:** 1 Department of Ophthalmology, The Second Affiliated Hospital of Harbin Medical University, Harbin, China; 2 The Key Laboratory of Myocardial Ischemia, Harbin Medical University, Ministry Education, Harbin, China; 3 Future Medical Laboratory, The Second Affiliated Hospital of Harbin Medical University, Harbin, China

**Keywords:** Neurotrophic factors, Haematopoietic stem cells

## Abstract

Glaucoma is characterized by retinal ganglion cell (RGC) death, the underlying mechanisms of which are still largely unknown. An E50K mutation in the Optineurin (OPTN) gene is a leading cause of normal-tension glaucoma (NTG), which directly affects RGCs in the absence of high intraocular pressure and causes severe glaucomatous symptoms in patients. Bone marrow (BM) stem cells have been demonstrated to play a key role in regenerating damaged tissue during ageing and disease through their trophic effects and homing capability. Here, we separated BM stem cells into Sca-1^+^ and Sca-1^-^ cells and transplanted them into lethally irradiated aged OPTN E50K mice to generate Sca-1^+^ and Sca-1^−^ chimaeras, respectively. After 3 months of BM repopulation, we investigated whether Sca-1^+^ cells maximized the regenerative effects in the retinas of NTG model mice with the OPTN E50K mutation. We found that the OPTN E50K mutation aggravated age-related deficiency of neurotrophic factors in both retinas and BM during NTG development, leading to retinal degeneration and BM dysfunction. Sca-1^+^ cells from young healthy mice had greater paracrine trophic effects than Sca-1^−^ cells and Sca-1^+^ cells from young OPTN E50K mice. In addition, Sca-1^+^ chimaeras demonstrated better visual functions than Sca-1^−^ chimaeras and untreated OPTN E50K mice. More Sca-1^+^ cells than Sca-1^−^ cells were recruited to repair damaged retinas and reverse visual impairment in NTG resulting from high expression levels of neurotrophic factors. These findings indicated that the Sca-1^+^ cells from young, healthy mice may have exhibited an enhanced ability to repair retinal degeneration in NTG because of their excellent neurotrophic capability.

## Introduction

Glaucoma is the most frequent cause of irreversible blindness worldwide, and its prevalence is increasing [1]. It develops from a complex interaction of multiple factors, including high intraocular pressure (IOP), advanced age, and genetic mutations, and is characterized by progressive degeneration and loss of retinal ganglion cells (RGCs). At present, the most commonly practiced therapy for glaucoma in the clinic is the reduction of IOP, but this strategy is unable to restore damaged RGCs and protect against blindness [2, 3]. In addition, a large population of patients, especially in Asia, have IOP in the normal range; glaucoma in such patients is called normal-tension glaucoma (NTG) [4, 5]. Neuroprotection has become a promising alternative approach to promote retinal cell survival by enhancing resistance to degenerative processes and pathological insult, which may prevent or reverse vision loss.

Bone marrow (BM) stem cells are ideal cells for retinal degeneration disease therapy and can be mobilized in response to tissue injury and repair to exert paracrine trophic effects [6, 7]. The E50K mutation of the Optineurin (OPTN) gene is known to cause NTG along with severe clinical glaucomatous symptoms [4, 8]. Thus, we established OPTN E50K mice by CRISPR-Cas9 gene-editing technology to investigate the role of BM stem cells in NTG treatment. For optimal healing capacity, young BM stem cells are preferred as donor cells for BM reconstitution in old recipients since ageing diminishes stem cell function [9]. Recent research, including our previous research, has found that young BM stem cells expressing antigen 1 (Sca-1^+^), which is widely used as a marker to isolate hematopoietic stem cells, have the greater homing and regenerative ability for aged or injured organisms than cells not expressing this antigen (Sca-1^−^) [10, 11]. Thus, young BM-derived Sca-1^+^ cells were used to reconstitute old OPTN E50K mice to investigate the important role of NTG in retinal neuroprotection and the underlying mechanism.

In this study, we advanced the understanding of the pathogenesis of NTG with quantitative proteomic analysis and demonstrated that young healthy Sca-1^+^ cells can maximize the regenerative effects on NTG retinas because of their excellent neurotrophic capability.

## Results

### Role of neurotrophic factors in NTG pathogenesis

**Fig. 1:**
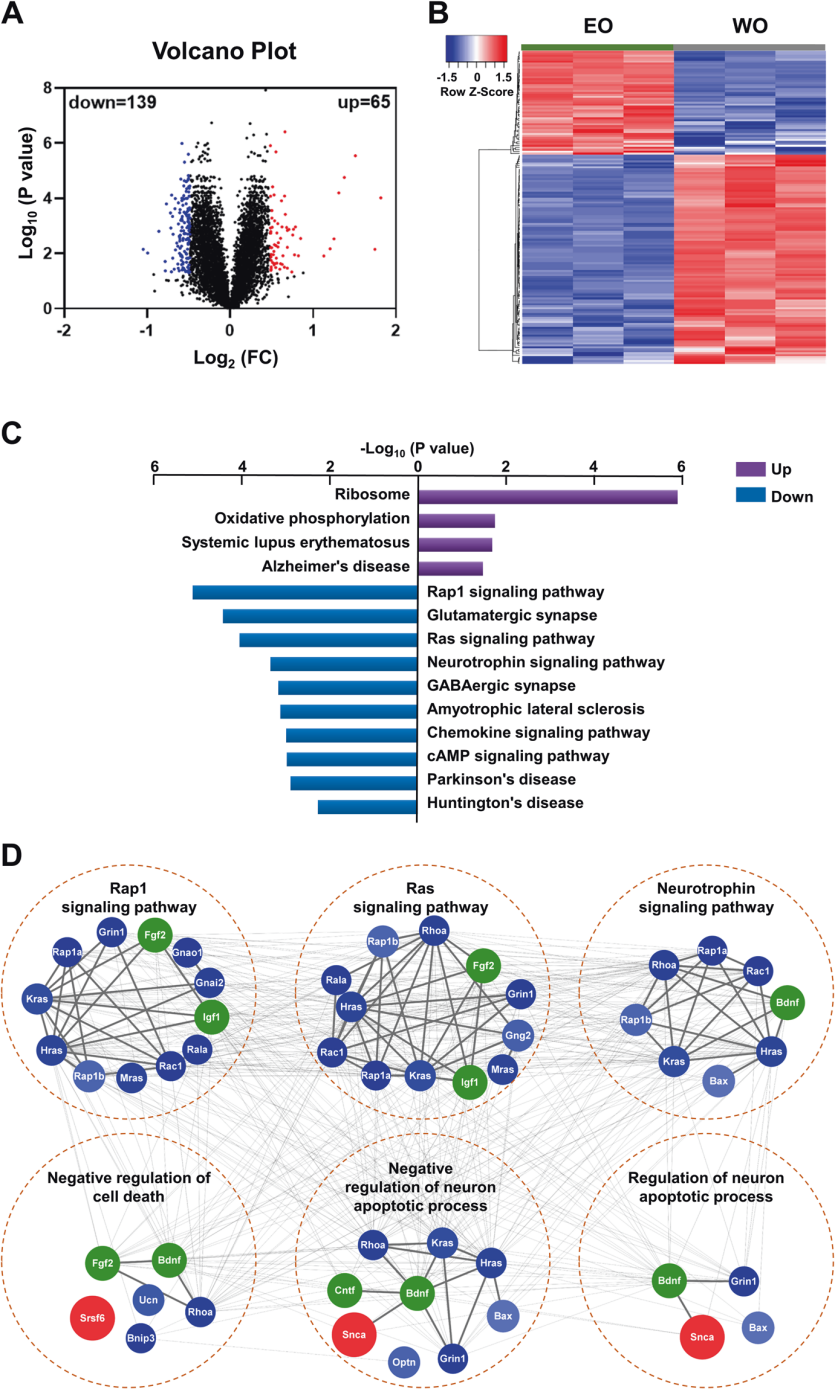
Proteomics of old WT and OPTN E50K retinas. **A** Volcano plot demonstrating the dual thresholds for differentially expressed proteins (DEPs) in old E50K (EO) mice versus old (18 months) wild-type (WO) mice. Each dot represents a single quantified protein. The dots with color in the upper and outer quadrants represent DEPs, with blue indicating a relative decrease and red indicating a relative increase in abundance (FC ≥ 1.40 or ≤0.71, *P* < *0.05*). **B** Hierarchical clustering heatmap depicting individual samples and gene expression differences between EO and WO samples (*n* = 3 per group). **C** Bar chart of the DEPs organized by enriched KEGG pathways as determined with the Database for Annotation, Visualization, and Integrated Discovery (DAVID). **D** Network generated with proteins associated with the significantly enriched pathways and biological processes and with NFs (green). The blue and red colors represent the upregulated and downregulated proteins, respectively.

Previous studies have demonstrated that deficits in neurotrophic factors (NFs) are hallmarks of senescence and neurodegenerative diseases, including glaucoma [12]. To investigate the underlying pathogenesis of NTG, we performed TMT-labeled LC-MS/MS proteomics analysis on old OPTN E50K and WT control mouse retinas (Fig. 1). The distribution patterns of enriched proteins within the EO and WO groups were assessed with volcano plots of log-transformed protein abundance ratios (Fig. 1A), and 204 differentially expressed proteins (65 upregulated, 139 downregulated; FC ≥ 1.40 or ≤0.71, *P* < 0.05) were found. The distribution patterns of these proteins in triplicate samples belonging to the WO and EO groups are displayed in a heat map (Fig. 1B). Then, the proteins were annotated with the DAVID annotation tool using the complete mouse proteome as the background. The downregulated proteins were significantly associated with a variety of neurodegenerative diseases, as well as certain important cellular pathways, such as neurotrophin signaling pathways (Fig. 1C). In addition, the NFs, brain-derived neurotrophic factor (BDNF), ciliary neurotrophic factor (CNTF), fibroblast growth factor 2 (FGF2), and insulin-like growth factor 1(IGF-1) also participated in the Rap1 and Ras signaling pathways; they were significantly enriched in the KEGG pathway and the biological process of neural apoptosis (Fig. 1D).

**Fig. 2:**
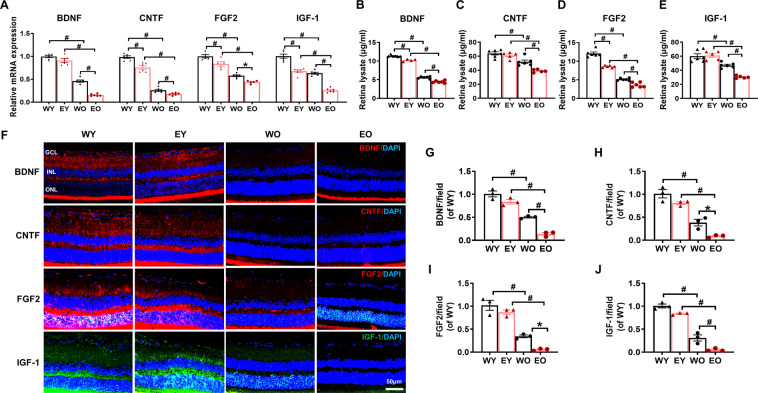
OPTN E50K mutation aggravated age-related neurotrophic factor deficiency in retinas. **A** The mRNA levels of neurotrophic factors (NFs) were determined by real-time qPCR in both young and old wild-type (WT) and OPTN E50K mutant (E50K) mice. The mRNA expression of brain-derived neurotrophic factor (BDNF), ciliary neurotrophic factor (CNTF), fibroblast growth factor 2 (FGF2), and insulin-like growth factor 1(IGF-1) significantly decreased with age and was most dramatically decreased in EO retinas. WY: young WT; EY: young E50K. **B**–**E** The protein expression of BDNF, CNTF, FGF2, and IGF-1 in the retinas of the four groups was determined by ELISA. The protein levels of these NFs significantly decreased with age and were most dramatically decreased in the EO group (*n* = 4–6/group). **F**–**J** Retinal sections were immunostained for BDNF, CNTF, FGF2, and IGF-1 in all 4 groups. The cell nuclei were visualized with DAPI. The intensity of staining of these NFs was significantly lower in the EO group than in the other 3 groups. Scale bar: 50 µm (*n* = 3/group). The data are shown as the mean ± SEM. *P < 0.05, ^#^*P* < 0.01.

We then performed qPCR and ELISA to determine the mRNA (Fig. 2A) and protein (Fig. 2B–E) levels of the selected NFs in the WY, EY, WO, and EO groups. The expression of the NFs was greatly decreased with respect to ageing and mutation and was particularly reduced in EO retinas. Immunostaining analysis also confirmed that the disease-specific declines in NF levels spread across the whole retina (Fig. 2F–J). These results provide evidence that the OPTN E50K mutation may advance and aggravate age-related retinal NF deficiency, thereby having lasting and severe impacts related to visual impairment in NTG.

### OPTN E50K mutation-dependent effects on the bone marrow

**Fig. 3:**
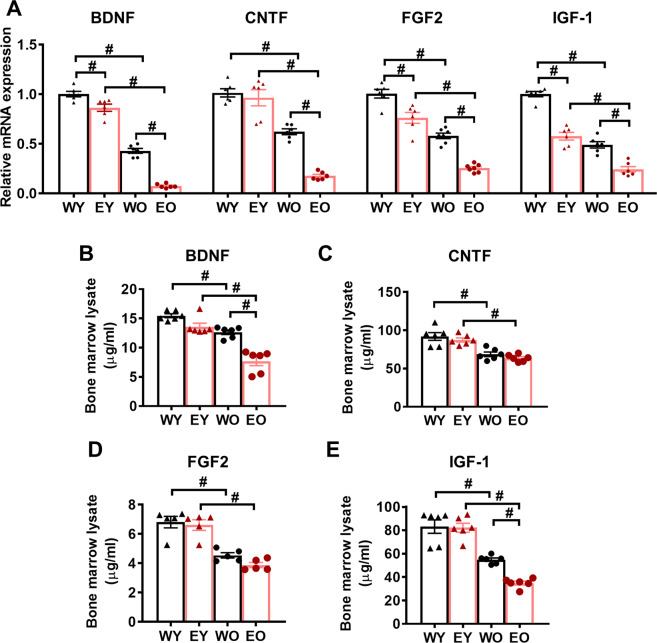
OPTN E50K mutation aggravated age-related neurotrophic factor deficiency in the bone marrow. **A** The mRNA levels of NFs were determined by real-time qPCR in the bone marrow (BM) of the four groups. The mRNA expression of BDNF, CNTF, FGF2, and IGF-1 significantly decreased with age and was most obviously decreased in EO mice. **B**–**E** The protein expression of BDNF, CNTF, FGF2, and IGF-1 in the BM of the four groups was determined by ELISA. The protein levels of these NFs were significantly lower in the EO group than in the other 3 groups. (*n* = 5–6/group). The data are shown as the mean ± SEM. *P < 0.05, ^#^*P* < 0.01.

Among tissues, BM contains the highest concentration of adult stem cells, which can be recruited to injured tissues and participate in regeneration with trophic effects [6]. However, this function may become blunted with age [9]. Therefore, we aimed to determine whether the stem cells in the BM of OPTN E50K mice had alterations in NF expression similar to those in the retinas. mRNA (Fig. 3A) and protein (Fig. 3B–E) expression levels in the BM of the four groups were measured. Interestingly, remarkable decreases in BDNF, CNTF, FGF2, and IGF-1 levels were also found in the old groups, particularly in the EO group, suggesting that the OPTN E50K mutation also diminishes the function of stem cells in the bone marrow.

### Trophic effects of young and healthy BM-derived Sca-1^+^ cells

**Fig. 4:**
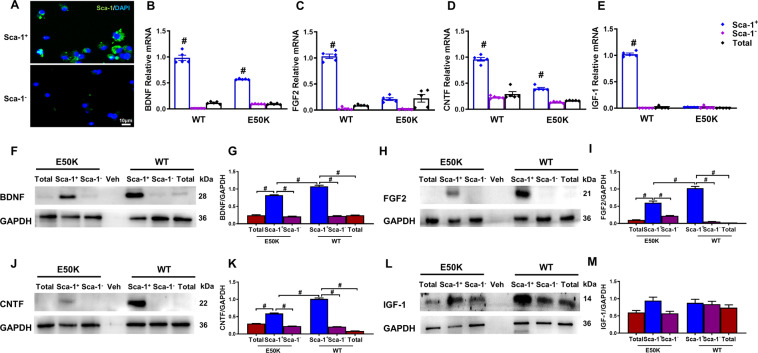
Differential expression profiles of neurotrophic factors between BM-derived Sca-1^+^ and Sca-1^-^ cells of two genotypes. **A** Bone marrow (BM)-derived Sca-1^+^ and Sca-1^−^ cells were immunostained for Sca-1 to confirm the phenotype. **B**–**E** The mRNA expression of NFs was determined by qPCR. The mRNA levels of BDNF, FGF2, CNTF, and IGF-1 were dramatically higher in Sca-1^+^ cells from young WT mouse BM than in Sca-1^−^ and total BM cells or Sca-1^+^ cells from young E50K mouse BM. Representative Western blot images and quantification of BDNF (**F, G**), FGF2 (**H, I**), CNTF (**J, K**), and IGF-1 (**L, M**) from Sca-1^+^, Sca-1^−^ and total cells from both young WT and E50K mouse bone marrow. The reagent only (Veh) was used for the control group. GAPDH was used as a loading control (*n* = 3/group). The data are shown as the mean ± SEM. *P < *0.05*, ^#^*P* < *0.01*.

Recent studies, including our previous report, have demonstrated that young BM-derived Sca-1^+^ cells have a strong ability to secrete cell factors [10, 13], which are responsible for protecting aged organisms and injured retinas. However, it has remained unknown whether the enhanced capabilities of Sca-1^+^ cells are affected by the OPTN E50K mutation as well considering our previous observations. Therefore, we isolated BM-derived Sca-1^+^ cells, Sca-1^−^ cells, and total cells from WT and OPTN E50K mice (Fig. 4A). We found that young Sca-1^+^ cells of both genotypes had higher mRNA expression of these NFs than old Sca-1^+^ and Sca-1^−^ cells (Supplemental Fig. 1A, B). We then assessed mRNA (Fig. 4B–E) and protein (Fig. 4F–M) expression of NFs in Sca-1^+^ cells compared with Sca-1^−^ and total cells in young WT and OPTN E50K mice to determine the E50K mutation-specific effects on BM. The results showed that Sca-1^+^ cells from WT mice had higher expression of BDNF, FGF2, CNTF, and IGF-1 than E50K-Sca-1^+^ cells and the cells in other groups, suggesting that the mutation reduced the characteristic trophic capabilities of Sca-1^+^ cells at an early age.

### Neuroprotective effects of Sca-1^+^ cells on cocultured old OPTN E50K retinas

**Fig. 5:**
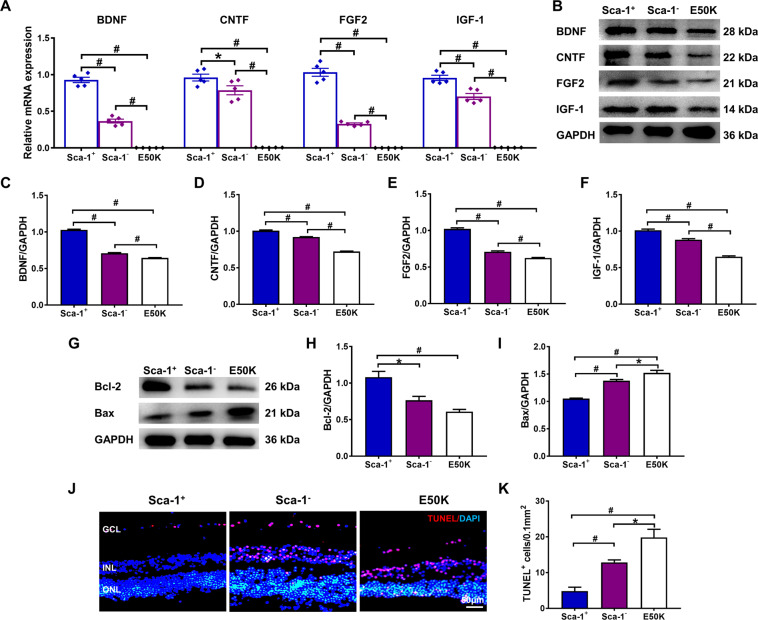
BM-derived Sca-1^+^ cells protected aged E50K retinal explants from apoptosis in vitro through the high expression of neurotrophic factors. Retinal explants from old E50K mice were cultured with young BM-derived Sca-1^+^ and Sca-1^-^ cells of WT mice, and untreated E50K retinal explants (E50K) acted as the control group. The mRNA and protein expression of BDNF, CNTF, FGF2, and IGF-1 was determined by qPCR (**A**) and Western blot analysis (**B-F**) in old E50K retinal explants cocultured with Sca-1^+^ and Sca-1^-^ cells. Representative Western blot images (**G**) and the relative amounts of Bcl-2 (**H**) and Bax (**I**) are shown. **J**, **K** Retinal sections were immunostained for TUNEL in the 3 groups. The cell nuclei were visualized with DAPI. The number of TUNEL^+^ cells in the ganglion cell layer (GCL) was significantly decreased in the Sca-1^+^ cell group (*n* = 3/group). The data are shown as the mean ± SEM. *P < *0.05*, ^#^*P* < *0.01*.

We utilized an in vitro coculture method to confirm whether Sca-1^+^ cells could protect NTG retinas from apoptosis through the neuroprotective effects of NFs. BM-derived Sca-1^+^ or Sca-1^−^ cells from young WT mice were cocultured on the inner surfaces of retinal explants of old OPTN E50K mice for 48 h and compared to retinal explants cultured with medium only as of the E50K group. The retinal explants for the three groups were then collected, and the mRNA and protein levels of NFs were assessed by qPCR (Fig. 5A) and Western blot analysis (Fig. 5B–F). The results demonstrated that both the mRNA and protein expression of BDNF, CNTF, FGF2, and IGF-1 in retinas was significantly increased after coculture with Sca-1 cells, particularly the Sca-1^+^ subset. We thus performed Western blot and TUNEL staining to assess retinal apoptosis. Consistent with the high levels of NFs, the protein expression of Bcl-2 was increased in the Sca-1^+^ group, while that of Bax was decreased (Fig. 5G–I). Quantification of apoptosis within the retinal explant RGC layer further confirmed that NTG-induced apoptosis was significantly attenuated by Sca-1^+^ cells compared to Sca-1^−^ and control groups (Fig. 5J, K).

### BM-derived Sca-1^+^ cells attenuated retinal degeneration in OPTN E50K mice

**Fig. 6:**
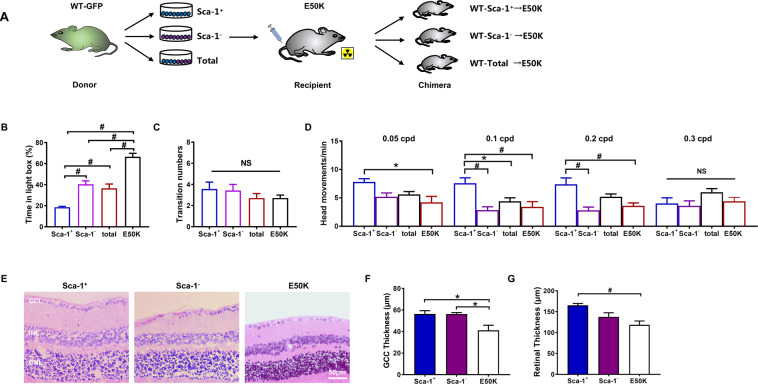
BM-derived Sca-1^+^ cells improved the visual condition of old E50K mice. **A** Schematic diagram showing the transplantation of old E50K mouse recipients with different subsets of bone marrow (BM). The BM of irradiated old E50K mice was reconstituted with young Sca-1^+^ cells, Sca-1^−^ cells, or unsorted total cells from WT mice to generate Sca-1^+^, Sca-1^−^ and total chimaeras, respectively. Light/dark exploration (**B, C**) and optomotor (**D**) tests revealed better preservation of visual behavior in chimaeras than in age-matched E50K mice, and the Sca-1^+^ chimaera group had the best preservation of visual behavior among these groups (*n* = 5–7/group). **E**–**G** H&E staining was performed to determine the ganglion cell complex (GCC) and retinal thicknesses in the Sca-1^+^ and Sca-1^−^ chimaera groups and the old E50K control group (*n* = 4/group). The data are shown as the mean ± SEM. *P < 0.05, ^#^*P* < 0.01.

To determine whether BM-derived Sca-1^+^ cells could home to the retina, repair glaucomatous damage, and improve visual function, we generated Sca-1^+^ (WT [Sca-1^+^]-E50K), Sca-1^-^ (WT [Sca-1^-^]-E50K) and total (WT [total]-E50K) chimaeras using young WT-GFP BM cells and lethally irradiated old OPTN E50K mice (Fig. 6A). There were no significant differences in physical condition variables, including appearance and body weight, between chimaeras and old OPTN E50K mice. After 3 months of reconstitution, the visual behavior of Sca-1^+^, Sca-1^−^ and total-BM chimaeras was evaluated using light/dark box exploration and optomotor response tests and compared to that of untreated age-matched OPTN E50K mice. In the light/dark exploration test, mice with normal visual behavior preferred the dark environment, and the time spent in the light compartment significantly increased with ageing and disease occurrence [10, 14]. After BM transplantation, the mice in all three chimaera groups spent less time in the light compartment than the old OPTN E50K mice (baseline), though no significant increases in transition numbers were observed (Fig. 6B, C). In addition, the time spent in the light compartment was significantly less for Sca-1^+^ chimaeras than for total and Sca-1^-^ chimaeras. The optomotor test was used to assess the quantifiable head movements in photopic conditions during the rotation of the grating. Although visual function was significantly decreased in old OPTN E50K mice, Sca-1^+^ chimaeras responded to lower frequencies with a greater number of head movements than the mice in the other groups. The total and Sca-1^−^ chimaeras showed no obvious differences from untreated OPTN E50K mice (Fig. 6D). These results indicated that the visual function of old OPTN E50K mice was improved after BM transplantation with Sca-1^+^ cells. The retinal morphology of Sca-1^+^ and Sca-1^−^ chimaeras was compared to that of untreated OPTN E50K retinas using hematoxylin and eosin staining (Fig. 6E–G). We found that the retinal ganglion cell complex (GCC) in Sca-1^−^ chimaeras were thicker than that in the untreated mice, and in Sca-1^+^ chimaeras, the whole retinas and GCCs were thicker than those in the untreated OPTN E50K mice. When we compared Sca-1^+^ chimaeras to old WT mice, we observed no significant differences between the two groups in visual behavior (Supplemental Fig. 2A, B), retinal morphology (Supplemental Fig. 2C, D) and RGC numbers (Supplemental Fig. 2E, F), suggesting that the visual condition of old OPTN E50K mice following Sca-1^+^ cell treatment was promisingly improved to a level similar to that in age-matched WT mice.

### BM-derived Sca-1^+^ cells had strong neuroprotective effects against NTG

**Fig. 7:**
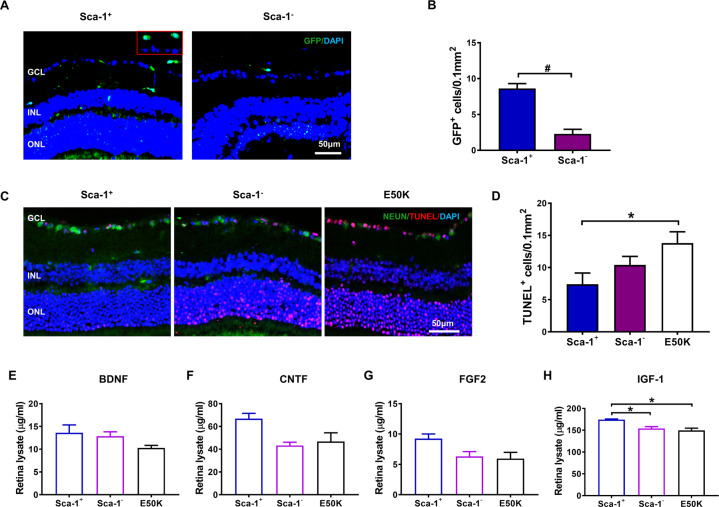
BM-derived Sca-1^+^ cells had enhanced homing and neuroprotection capability. BM-derived Sca-1^+^ or Sca-1^−^ cells from young GFP (green fluorescent protein, green) transgenic mice were used to reconstitute old E50K mice, generating Sca-1^+^ and Sca-1^-^ chimaeras, respectively. **A**, **B** Retinal sections of Sca-1^+^ and Sca-1^−^ chimaeras were immunostained for GFP to confirm the capability of BM-derived Sca-1^+^ or Sca-1^−^ cells to home to retinas. **C**, **D** Retinal sections were immunostained with TUNEL (red) and for NeuN (green) in the 3 groups. The cell nuclei were visualized with DAPI. The number of TUNEL^+^ cells in the GCL was significantly decreased in Sca-1^+^ chimaeras (*n* = 3/group). **E**–**H** The protein levels of BDNF, CNTF, FGF2, and IGF-1 in the retinas of Sca-1^+^ and Sca-1^−^ chimaeras and age-matched E50K mice were determined by ELISA (*n* = 3/group). The data are shown as the mean ± SEM. *P < 0.05, ^#^*P* < 0.01.

To further elucidate the underlying mechanisms responsible for repairing damaged retinas in chimaeric mice, immunostaining for GFP was performed to determine the homing capacity of young BM-derived Sca-1^+^ or Sca-1^−^ cells to recipient mouse retinas (Fig. 7A, B). Donor-derived GFP^+^ cells were found in the inner layers of the retinas of both Sca-1^+^ and Sca-1^−^ chimaeras; however, Sca-1^+^ cells had greater homing capability than Sca-1^−^ cells. In line with that, there were fewer apoptotic cells in the RGC layer in Sca-1^+^ chimaeras than in old OPTN E50K mice (Fig. 7C, D). Similarly, we also examined NF expression in the retinas of both chimaeras by ELISA (Fig. 7E–H). The protein expression of IGF-1 was explicitly increased in the retinas of Sca-1^+^ chimaeras compared to those of Sca-1^-^ chimaeras and OPTN E50K controls. The protein levels of BDNF, CNTF, and FGF2 in the Sca-1^+^ chimaera group were visibly higher than those in the other two groups, although the P-value did not meet the significance criteria. To further elucidate the role of BM-derived Sca-1 cells in vivo, we also examined the protein expression of these NFs in the bone marrow of all three groups, finding superior paracrine capability in Sca-1^+^ chimaeras (Supplemental Fig. 3A–D). Collectively, these results suggested that increased levels of the NFs BDNF, CNTF, FGF2, and IGF-1 may play a major role in mediating the retinal neuroprotective effect of BM-derived Sca-1 cells against NTG.

## Discussion

The pathological mechanism of glaucoma is still obscure. In this study, we demonstrated an age-related deficiency of NFs in both retinal and BM stem cells, which was aggravated by the OPTN E50K mutation during NTG development. These findings, supported by our proteomic analysis results, suggest the importance of NF deficits in NTG pathogenesis and suggest that BM dysfunction could contribute to retinal impairment during disease. A subset of BM-derived Sca-1 cells possessed superior trophic effects with high NF levels, and the cells were confirmed to alleviate retinal apoptosis and reverse visual loss in old OPTN E50K mice both in vitro and in vivo.

The OPTN E50K mutation is a common cause of NTG, in which RGCs are affected without high IOP and which causes severe symptoms in patients [4, 8]. Thus, we established OPTN E50K mice by CRISPR-Cas9 to model NTG in vivo, aiming to gain a more detailed understanding of the pathological mechanism in the context of ageing and to explore new viable therapies. Recent studies have postulated that trophic factor withdrawal, genetic determinants, and defective axon transport contribute to the pathologic process of glaucoma [15]. NFs, including BDNF, CNTF, FGF2, and IGF-1, are crucial to nervous system health maintenance during ageing. They can improve RGC survival and induce beneficial synaptic changes in experimental models of glaucoma [16–20] (Supplemental Fig. 4). Various preclinical studies have been conducted using these factors to treat glaucoma through intravitreal administration. The OPTN E50K mutation is reported to induce BDNF deficiency [21, 22]. Our proteomic analysis further provided evidence for the underlying relationship between NFs and NTG development. For the downregulated proteins, the Rap1, Ras, and neurotrophin signaling pathways and the neural apoptotic process were significantly enriched according to the DAVID database, in which BDNF, CNTF, FGF2, and IGF-1 were also involved. The retinal mRNA and protein levels of these trophic factors in our study were consistent with deficiency during ageing and disease. This is in agreement with recent findings that local delivery of neurotrophins was beneficial in a glaucoma model and may partly explain the enhancement of RGC survival by combined administration of trophic factors [23]. However, the safety of frequent intravitreal injection and challenges in maintaining protein stability in the eyes deserve serious consideration. In this context, advances in bone marrow-derived stem cell (BMSC) research has provided a rational option to treat retinal dysfunction.

Among tissues, bone marrow contains the highest concentration of adult stem cells. BMSCs are easily harvested and administered through intravenous injection [6]. Furthermore, they can repopulate the bone marrow in transplant recipients and can home to and repair degenerating or ischemic retinas through the systemic circulation [6, 10, 24]. Most retinal disorders involve damage to more than one cell type in the retinas and cause extensive remodeling [6]. Thus, the primarily paracrine trophic effects of BMSCs seem to be more viable than the effects of replacement of single specific cell types for retinal dysfunction treatment, especially given the NF deficiency observed in NTG. However, ageing can weaken the functional capacity of BMSCs [25] and aggravate various pathologic processes in old-age glaucoma with the E50K mutation. In our study, analysis of the expression of four selected trophic factors confirmed the occurrence of a similar age-driven deficit in NFs in retinal tissue as in bone marrow, suggesting that the critical regenerative capability of stem cells was also diminished.

Recent research and our previous work have revealed that a subclass of young BM-derived cells, Sca-1^+^ cells, enhance paracrine support and regenerative capability in aged retinas [10, 24]. We isolated Sca-1^+^ cells from young and old WT and OPTN E50K BM to assess their secretory capability. The results verified that these cells had greater paracrine trophic effects, which were affected by age in both genotypes. Unfortunately, the advantage of paracrine trophic effects in Sca-1^+^ cells at an early stage was reduced in mutants compared to WT controls. To maximize the potential therapeutic effect, we harvested BM-derived Sca-1^+^ cells from young WT mice for NTG treatment to compensate for NF declines in both the retinas and BM. We first cocultured aged OPTN E50K retinas with BM-derived Sca-1^+^ cells from young WT mice to investigate the therapeutic effects in vitro. Sca-1^+^ cells were observed to significantly promote RGC survival by exerting paracrine trophic effects superior to those of Sca-1^-^ cells. In addition to the increases in BDNF, CNTF, FGF2, and IGF-1 expression in cocultured retinas, downregulation of the apoptotic protein Bax was observed, and upregulation of the antiapoptotic protein Bcl2 was observed. In addition, the number of TUNEL-positive cells was obviously reduced in whole retinas, not only in the RGC layer, which verified that BMSCs were more universally applicable than a single type of cell owing to the multiple types of damaged retinal cells. Retinal and optic nerve damage in glaucoma is markedly worse in older individuals than in younger individuals; thus, we chose old OPTN E50K mice as recipients and conducted BM transplantation to establish Sca-1^+^ chimaeras in order to examine the superior repair function on visual impairment in NTG. We observed enhanced mobilization of BM-derived Sca-1^+^ cells in the host retinas, and the homed Sca-1^+^ cells protected RGCs from apoptosis through increased BDNF, CNTF, FGF2, and IGF-1 expression. Higher NF concentrations are linked to higher stress resilience, which is beneficial for neuronal cells affected by the disease. We also found that the aged BMSCs in OPTN E50K mice were rejuvenated such that they secreted more trophic factors after reconstitution with young Sca-1^+^ cells. A series of experiments further confirmed that these responses could alleviate visual impairment in NTG [26]. As reported in previous studies, mice with normal sight showed aversion to light in the light/dark exploration behavior test [14, 27]. Here, we demonstrated that compared to the untreated control mice, old OPTN E50K mice receiving BM transplantation of Sca-1^+^, Sca-1^−^ and total stem cells all showed robust aversion to light, although their movements were not significantly different from those of the control mice. Accordingly, the Sca-1^+^ group showed the most notable change among the three chimaera groups. These results were consistent with the optomotor response test results. Sca-1^−^ and total chimaeric old OPTN E50K mice failed to exhibit improvements in retinal degeneration, displaying similar optomotor responses as the untreated mice with the same spatial frequencies in the grating test. The Sca-1^+^ chimaeras still exhibited more active head responses from 0.05 to 0.2 cpd under NTG conditions. Sca-1^+^ cells also blocked retinal thinning in NTG. The improvement in the visual condition of Sca-1^+^ chimaeras was similar to that of age-matched WT mice. There was no significant difference between the groups in retinal function and morphology, suggesting promising regenerative capability of Sca-1^+^ cells. Thus far, many studies have highlighted the pathological similarities between glaucoma and neurodegenerative diseases in the central nervous system [28–30], as supported by our proteomic analysis. Retinas respond similarly to the brain under pathological conditions since they are extensions of the CNS [31, 32]. Given the neuroprotective role of Sca-1^+^ cells in NTG, this study provides preliminary evidence for a new potential therapy to mitigate the effects of age-related neurodegenerative diseases.

## Conclusion

Our study elucidated that the OPTN E50K mutation was associated with aggravation of age-related deficiency of NFs in both retinas and bone marrow, which may have led to retinal degeneration and reduced regenerative function in BM during NTG development. We also found that young BM-derived Sca-1^+^ cells had primarily paracrine trophic effects and were able to rescue damaged RGCs in vitro. We further outlined the possibility of using Sca-1^+^ cells to reverse visual impairment in NTG via their superior homing capability and paracrine trophic effects on old OPTN E50K mice in vivo. These findings shed light on the pathological mechanism of NTG and the neuroprotective effects of BM-Sca-1^+^ cells, which could aid in understanding the process of NTG and facilitate the development of new viable therapies for degenerative diseases.

## Methods

### Animals and mouse model of NTG

All experimental protocols were approved by the Institutional Animal Care and Use Committee at Harbin Medical University and conformed to the Guide for the Care and Use of Laboratory Animals (NIH, 8th Edition, 2011) and the guidelines of the Ethics Committee of the Second Affiliated Hospital of Harbin Medical University (Permit Number: KY 2018-220). OPTN E50K mice were established by CRISPR-Cas9 genome editing as an NTG mouse model, and age-matched wild-type (WT) mice served as the control group. The mice were housed in the specific pathogen-free animal facility at the Second Affiliated Hospital of Harbin Medical University. Groups of mice with the OPTN E50K mutation at 3 and 18 months were designated the EY and EO groups, respectively, while the age-matched WT control groups were correspondingly defined as the WY and WO groups. As previously described, the results of visual function tests and retinal morphology experiments showed that both WT and OPTN E50K mice demonstrated age-related visual impairment without IOP evaluation, while EO mice displayed more severe retinal degeneration than the other mice that was similar to the degeneration observed in clinical NTG [10, 33].

### Proteomics

To investigate the molecular characteristics of the NTG mouse model with the OPTN E50K mutation, we performed tandem mass tag quantitative proteomics in retinas and bioinformatics analysis to elucidate the underlying biochemical processes. Retinas were subjected to appropriate sample preparation methods for MS-based proteomics, including protein digestion, peptide labeling, fractionation, and MS analysis, and the raw data files obtained were processed with the MaxQuant search engine (v.1.5.2.8). DAVID Bioinformatics (version 6.8) was used to classify the differentially expressed proteins via Gene Ontology analysis, especially in the biological process category, and via Kyoto Encyclopedia of Genes and Genomes (KEGG) analysis [34]. Protein interaction networks were generated using the Cytoscape tool (http://apps.cytoscape.org).

### Quantitative real-time PCR (qPCR) analysis

Total RNA was extracted from retinas with TRIzol and converted into cDNA with a Transcription First Strand cDNA Synthesis Kit (Roche, UK). Real-time qPCR was carried out according to the manufacturer’s protocols with LightCycler 480 SYBR Green I Master Mix (Roche, UK). The mRNA expression of genes of interest was normalized to the expression GAPDH using the 2^−ΔΔct^ method.

### Western blot analysis

Dissected retinas were lysed for 30 min on ice with RIPA lysis buffer (CWBIO) and centrifuged (12,000 rpm) at 4 °C for 10 min. Proteins were then collected, diluted in SDS-PAGE loading buffer (Beyotime), and heated at 95 °C for 10 min. The concentration of proteins was assessed by the BCA method, and equal amounts of protein were electrophoresed on 10–15% SDS-polyacrylamide gels and then transferred onto polyvinylidene fluoride membranes. The membranes were then blocked with 5% skim milk solution for 1 h at room temperature before incubation with primary antibodies against BDNF (Proteintech, 28205), CNTF (Santa Cruz Biotechnology, 365210), FGF2 (Santa Cruz Biotechnology, 136255), IGF-1 (Abcam, 9572), Bcl-2 (Proteintech, 26593), and Bax (Proteintech, 50599) overnight at 4 °C. After three washes, the membranes were incubated with horseradish peroxidase-conjugated secondary antibodies (1:10,000, Zhongshan) for 1 h at room temperature. The protein bands were visualized by enhanced chemiluminescence (ECL, Biosharp) and quantified with ImageJ. The protein levels are expressed as the protein of interest/GAPDH ratios.

### Immunofluorescence staining

Mice were sacrificed and perfused with 4% paraformaldehyde. Eye tissues were fixed with 4% paraformaldehyde overnight at 4 °C, placed in a 25% sucrose solution for dehydration, and then frozen. The frozen eyes were embedded in the optimal cutting temperature compound and cut into 6 µm sections. After washing with PBS, the eye tissue sections were blocked in 0.5% goat serum for 1 h and then subsequently incubated with primary antibodies overnight at 4 °C. To examine RGC apoptosis, NeuN antibodies (Abcam, 177487) and TUNEL reagent (Roche, 12156792910) were used. The tissue sections were incubated with Alexa Fluor 488- or 594-conjugated secondary antibodies for 1 h at room temperature and then with DAPI for 5 min to counterstain the nuclei. Images were taken using a fluorescence microscope and quantified with Image-Pro software.

### Bone marrow transplantation

BM transplantation was performed as described previously [10]. In brief, young BM cells were harvested from C57BL/6-Tg-GFP mice by flushing the femurs and tibias with PBS. The cells were separated into Sca-1^+^ and Sca-1^−^ subsets by immune magnetic-activated cell sorting following the manufacturer’s instructions (Stem Cell Technology, Canada). Sca-1^+^, Sca-1^−^ or unsorted total BM cells (2 × 10^6^) were directly injected into lethally irradiated (8.5 Gy) recipient OPTN E50K mice at 18 months old through the tail vein to generate Sca-1^+^ (WT[Sca-1^+^]-E50K), Sca-1^−^ (WT[Sca-1^−^]-E50K) and total BM (WT[total]-E50K) chimaeras, respectively. Three months later, the chimaeric mice were subjected to further experiments.

### Enzyme-linked immunosorbent assays

Proteins were isolated from retinal tissue or bone marrow stem cells using RIPA buffer containing protease and phosphatase inhibitors. The amount of recovered protein was evaluated with a BCA kit (Suolaibao, China). The levels of mouse BDNF, CNTF, FGF2, and IGF-1 in retinal tissue and bone marrow stem cells were determined using the aforementioned enzyme-linked immunosorbent assay (ELISA) kits (Chenglin, China) according to the manufacturer’s instructions.

### Light/dark box exploration task

As previously described [10, 33], we investigated light-mediated visual behavior using a light/dark box exploration paradigm. The light/dark box consisted of a dark chamber and an illuminated chamber of equal size with an aperture located in the middle wall between the two chambers that allowed the mice to weave freely through the compartments. The wall of the light chamber was made with an LED screen, and the dark chamber was surrounded by light-impermeable fabric to ensure complete darkness. The light/dark test was based on rodents’ preference for darker compared to brighter areas, and the increase in the time that they stayed in the light chamber suggested diminished visual acuity. The mice were kept in complete darkness overnight for dark adaptation before testing and then directly placed into the dark chamber of the light/dark box. After the mice were allowed to acclimatize for a few minutes, a 10-min test was begun. We recorded the time the mice spent in the light chamber and the number of transitions between the two chambers. Each mouse was tested 3 times per trial, and the data were averaged for further analysis. Seven to ten animals from each experimental group were tested.

### Optomotor response task

Optomotor response measurement is a widely established method to assess visual function in animal models of disease. With increasing spatial frequency, there should be a threshold at which no meaningful head movements are detected. Normally, the highest spatial frequency below this threshold that elicits optomotor responses provides an estimate of visual acuity [10]. Thus, we examined the optomotor responses of our experimental mice to assess the visual impairment caused by NTG. In our optomotor response measurement equipment, both the bottom and the lid of the box were covered with a mirror. There was a platform in the center of the box, where the mice were placed during the test. A camera was located at the center of the lid and connected to a computer through USB to record mouse head movements. The box accommodated four LCD screens that surrounded the animal, and these screens were connected to the computer through DVI to enable control of the experiment. Vertical black and white stripes were presented on the screen to evoke the optomotor reflex in mice, and these stripes were rotated under a defined spatial frequency in each trial. Four spatial frequencies were used for the experiment, including 0.05, 0.1, 0.2, and 0.3 cycles/degree (cpd). After a few minutes of adaptation, the mice were tested at each frequency for 2 min with a 30-s interval between two rotations, and head movements corresponding to drum rotation were scored.

### Statistics

All values are presented as the mean ± SEM. Statistical analysis was conducted using Prism version 7.0 (GraphPad Software), and the results were analyzed by unpaired Student’s *t*-test or one-way ANOVA followed by Tukey’s post hoc test. *P* < *0.05* was considered to indicate statistical significance.

## Supplementary information

supplement figure1

supplement figure2

supplement figure3

supplement figure4

supplement figure legend Please help us correct this sentence ‘Retinal explants from old E50K mice were cultured with FGF2 (A-C, G, H) and IGF-1(D-F, I, J) for 1μg and 2μg respectively. Untreated E50K retinal explants (E50K) acted as control group.’in Supplemental Fig.4 to ‘Retinal explants from old E50K mice were cultured with FGF2 (A-C, G, H) andIGF-1(D-F, I, J) for 200ng/ml and 400ng/ml respectively. Untreated E50K retinal explants (0) acted as the controlgroup.’ Thanks a lot.
