# Supplementary figures and images for: Neuroprotective effects of bone marrow Sca-1+ cells against age-related retinal degeneration in OPTN E50K mice
Source: Cell Death Dis. 2021 Jun 15;12(6):613. doi: 10.1038/s41419-021-03851-0 (PMC8203676; doi:10.1038/s41419-021-03851-0)

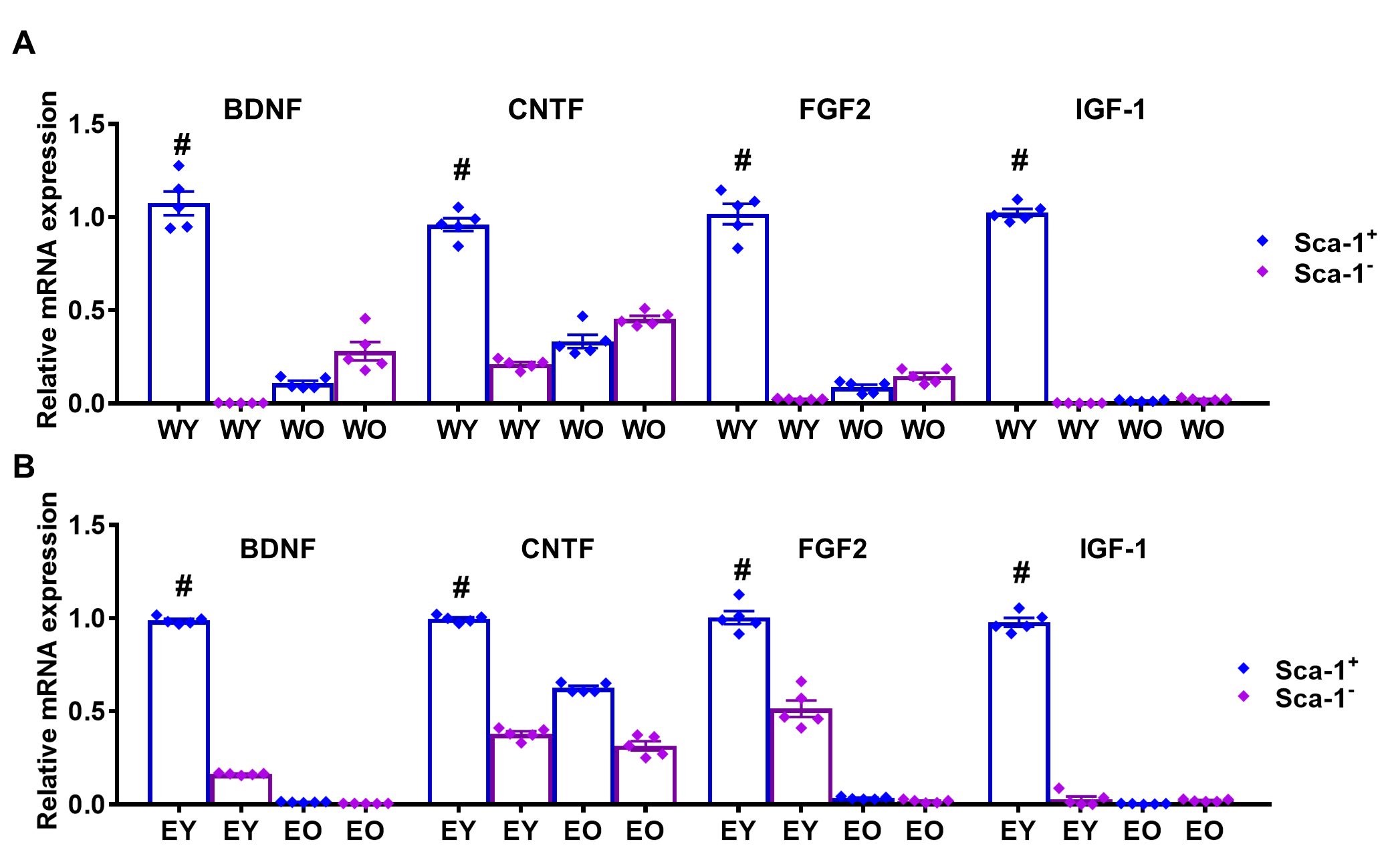

Supplement: Supplementary file 1 — supplement figure1 [file 41419_2021_3851_MOESM1_ESM.tif]

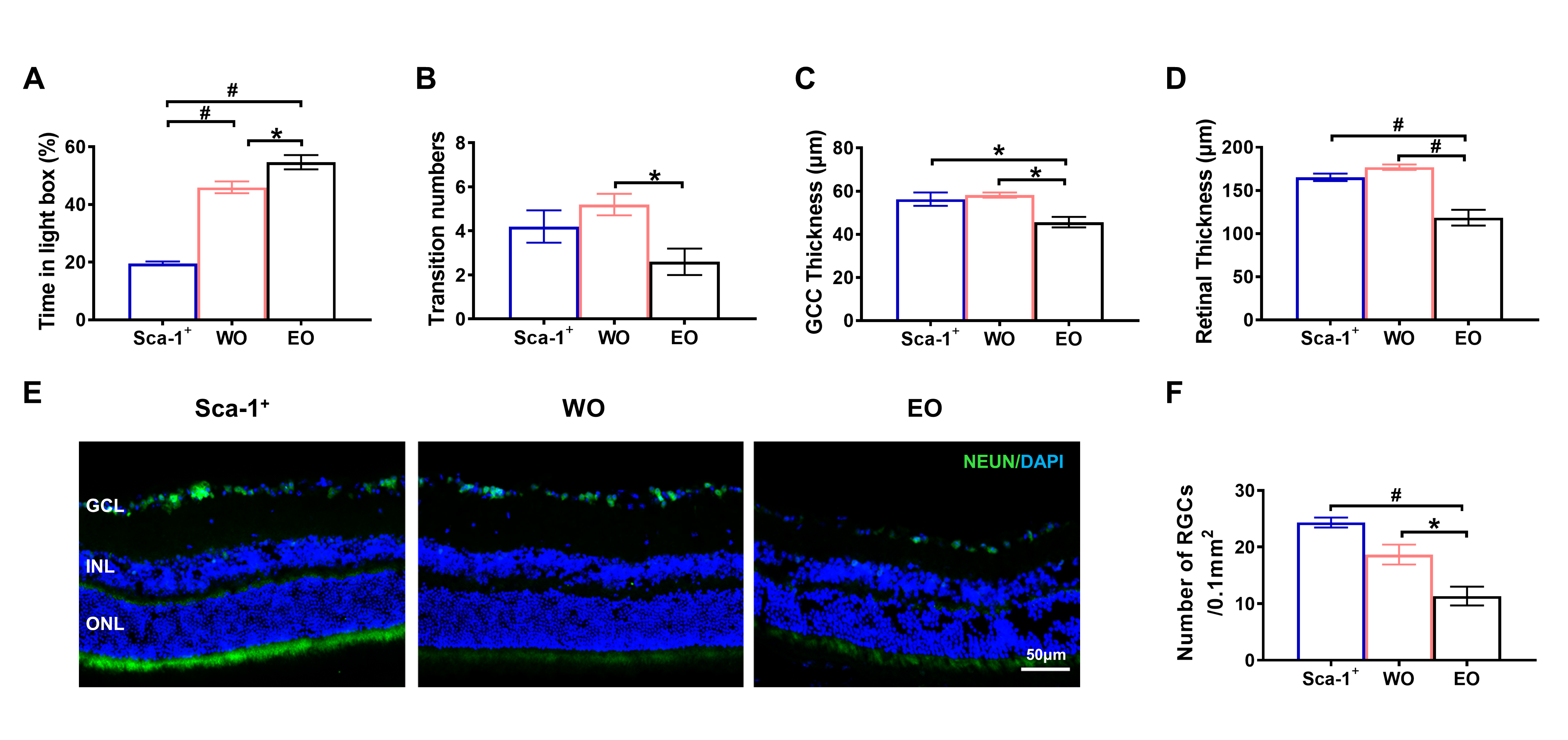

Supplement: Supplementary file 2 — supplement figure2 [file 41419_2021_3851_MOESM2_ESM.tif]

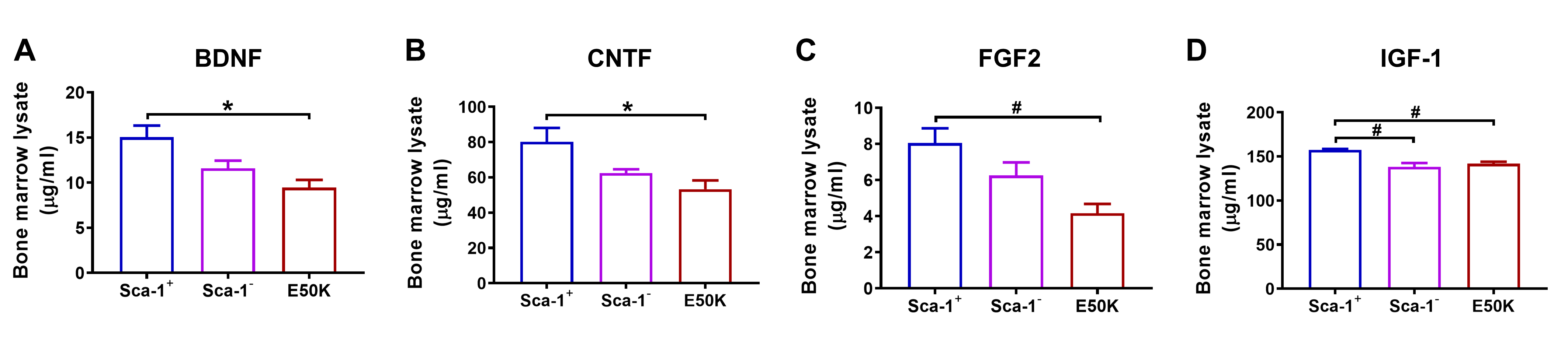

Supplement: Supplementary file 3 — supplement figure3 [file 41419_2021_3851_MOESM3_ESM.tif]

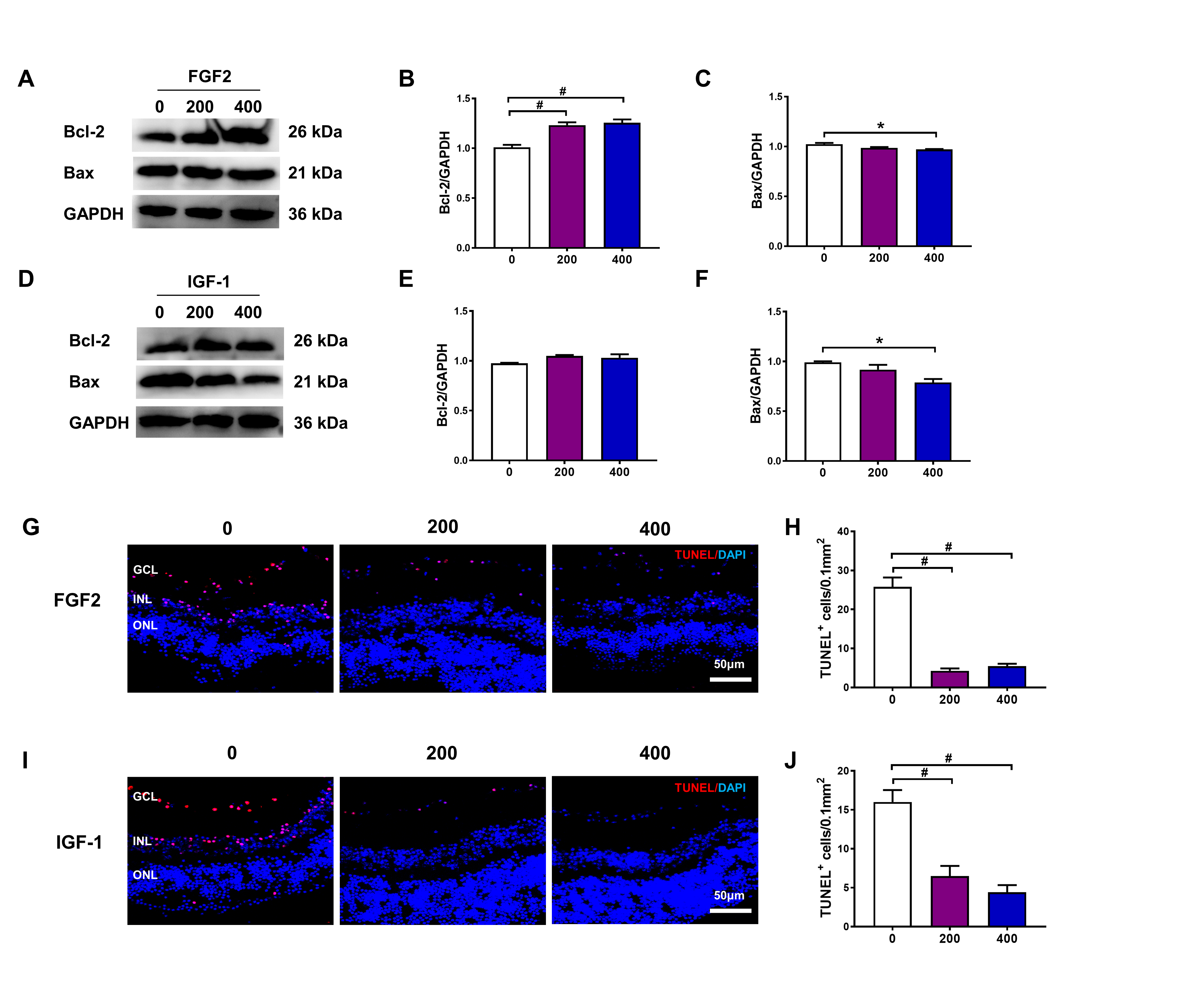

Supplement: Supplementary file 4 — supplement figure4 [file 41419_2021_3851_MOESM4_ESM.tif]
