# Supplementary material for: Neuroprotective effects of bone marrow Sca-1+ cells against age-related retinal degeneration in OPTN E50K mice
Source: Cell Death Dis. 2021 Jun 15;12(6):613. doi: 10.1038/s41419-021-03851-0 (PMC8203676; doi:10.1038/s41419-021-03851-0)
Supplement: Supplementary file 5 — supplement figure legend Please help us correct this sentence ‘Retinal explants from old E50K mice were cultured with FGF2 (A-C, G, H) and IGF-1(D-F, I, J) for 1μg and 2μg respectively. Untreated E50K retinal explants (E50K) acted as control group.’in Supplemental Fig.4 to ‘Retinal explants from old E50K mice were cultured with FGF2 (A-C, G, H) andIGF-1(D-F, I, J) for 200ng/ml and 400ng/ml respectively. Untreated E50K retinal explants (0) acted as the controlgroup.’ Thanks a lot. [file 41419_2021_3851_MOESM5_ESM.docx]

**Supplemental Fig.1 Differential expression profile of neurotrophic factors between young and old BM-derived Sca-1^+^ and Sca-1^-^ cells of both WT and E50K mice.**

The mRNA expression of BDNF, CNTF, FGF2 and IGF-1 in Sca-1^+^ cells from young bone marrow (BM) were significantly higher than Sca-1^-^ cells and Sca-1^+^ cells from old BM in both WT (**A**) and OPTN E50K mice (**B**). #, *P* < 0.01.

**Supplemental Fig. 2 Sca-1^+^ chimaeras shared similar visual condition with old WT mice.**

**A, B** The light/dark exploration tests revealed better preservation of visual behavior in Sca-1^+^ chimaeras and WO comparing to EO (n = 5/group). **C, D** Ganglion cell complex (GCC) and retinal thicknesses in Sca-1^+^ chimaeras, WO and EO groups (n = 3-4/group). **E, F** Retinal sections were immuno-stained for Neun in 3 groups. Cell nuclei were visualized with DAPI (n = 3/group). *, *P* < 0.05, #, *P* < 0.01.

**Supplemental Fig. 3** **BM stem cells in Sca-1^+^ chimaeras had greater trophic effects. A-D** The protein levels of BDNF, CNTF, FGF2 and IGF-1 in bone marrow of Sca-1^+^ chimaeras were significantly higher than age-matched E50K mice (n=3/group). Data shown as mean ± SEM. *P<0.05, #*P* < 0.01.

**Supplemental Fig.4 Neurotrophic factors,** **FGF2 and IGF-1, protected aged E50K retinal explants from apoptosis in vitro.**

Retinal explants from old E50K mice were cultured with FGF2 (**A-C, G, H**) and IGF-1(**D-F, I, J**) for 200ng/ml and 400ng/ml respectively. Untreated E50K retinal explants (0) acted as control group. Representative Western blot image and relative amounts of Bcl-2 and Bax for FGF2 (**A-C**) group and IGF-1 group (**D-F**). Retinal sections were immuno-stained for TUNEL in FGF2 group (**G, H)** and IGF-1 group (**I, J**). Cell nuclei were visualized with DAPI (n = 3/group). Data shown as mean ± SEM. **P<0.05*, #*P* < 0.01.
